# Computed Tomographic and Clinical Findings in Domestic Rabbits (*Oryctolagus cuniculus domesticus*) with Pulmonary Emphysema

**DOI:** 10.1111/vru.70063

**Published:** 2025-07-16

**Authors:** Athinodoros Athinodorou, Nicolas Israeliantz, Jenna Richardson, Dario Costanza, Jorge del Pozo, Tobias Schwarz

**Affiliations:** ^1^ Royal (Dick) School of Veterinary Studies and Roslin Institute The University of Edinburgh Easter Bush Estate Roslin UK; ^2^ Interdepartmental Centre of Veterinary Radiology University of Napoli “Federico II” Napoli Italy

**Keywords:** CT, lagomorph, lung, respiratory

## Abstract

Pulmonary emphysema (PE) is a poorly understood condition in rabbits. This retrospective case‐control study investigated the CT and clinical findings of rabbits with PE. Institutional archive review identified 724 thoracic CT studies of 529 rabbits, including 76 PE‐positive studies of 59/529 rabbits. Twenty‐five PE‐negative cases were selected randomly as a control group. The mean age of affected rabbits was 9 years (range 5–13 years). Cranial lung lobes were more commonly affected (*p* < .01). The X‐ray attenuation in Hounsfield units (HU) of the emphysematous lung areas (median −905 HU) was significantly lower than in nonemphysematous lung lobes of the case (median −667 HU) and control group (median −652 HU). There was significantly lower X‐ray attenuation in peripheral and bullous emphysema than in diffuse emphysema. There was no statistical correlation between clinical lower respiratory signs and PE presence. However, the small portion (*n* = 6, 10.2%) of affected rabbits with severe respiratory signs, such as open‐mouth breathing and cyanotic mucous membranes, all had advanced PE and poor outcome. Secondary changes attributable to PE included pathologic rib fractures in 3 (5.1%) and bulla rupture leading to pneumothorax in 2 (3.4%) rabbits. Of the 15 rabbits with repeat examinations, PE was progressive in 12 (80%) and static in 3 (20%). PE is a common condition in rabbits that is readily detectable with CT. The progressive nature of PE should be considered when detected in asymptomatic rabbits. In rabbits with severe lower respiratory signs, PE should be considered as a potential cause.

## 1 Introduction

Pulmonary emphysema (PE) is a perceived rare condition in domestic animals. It is characterized by collapse of the bronchial wall, bronchial obstruction, and air trapping in the pulmonary parenchyma [1, 2]. The prevalence of PE in humans is linked to exposure to irritants such as tobacco smoke and environmental pollutants, such as fumes and bacteria. [1, 3] A genetic link to the occurrence of congenital lobar emphysema exists in humans, dogs, and cats [1, 3, 4, 5, 6]. Pulmonary emphysema has been reported in dogs, cats, and horses [1, 4, 5, 7], but in domestic rabbits, it has only been reported in one case report and a small case series [8, 9]. In human medical research, rabbits have historically been utilized as experimental models for the study of lung disease [10, 11, 12, 13]. The etiology and pathophysiology of pulmonary emphysema have, therefore, only been reported in experimental environments [12, 13, 14].

In humans, cats, and dogs, the antemortem diagnosis of emphysematous lesions is mostly facilitated by CT, to a lesser extent by radiography or a combination of both [1, 2, 4, 5]. Definitive diagnosis can be achieved with histopathology [12, 14]. Lung lobectomy is often performed in cats and dogs for congenital emphysema, but is rarely performed in rabbits, mainly because of cost, the increased anesthetic risks, and postoperative complications, such as gastrointestinal stasis and acute death [2, 8].

This study aimed to investigate the CT and clinical findings of rabbits with pulmonary emphysema. We hypothesized that in rabbits, (1) PE is a common CT finding; (2) PE correlates with clinical signs of lower respiratory tract (LRT) disease; (3) the X‐ray attenuation of emphysematous lung lobes is visibly and measurably reduced compared with normal lungs; and (4) PE is progressive on sequential CT examinations.

## 2 Materials and Methods

### 2.1 Study Population

This was a single‐center, retrospective case and control study with specific analytical components, with ethical institutional approval (Veterinary Ethical Review Committee reference 132.21).

Diagnostic CT studies of rabbits conducted at the Royal (Dick) School of Veterinary Studies between 2017 and 2023 were assessed, along with patient medical records, enabling comprehensive clinical data collection. Inclusion criteria for the case and control group included a diagnostic quality CT study of the thorax of a domestic rabbit performed with a standardized protocol and minimal signalment data (age, breed, sex, neutered status, body weight). Collected clinical data included reason for investigation, presenting clinical signs, information on husbandry, exposure to environmental pollutants, and thoracic trauma. Data collection for the case group referred to the dates of the first CT examination with evidence of PE between 2017 and 2023 and the last available CT examination between 2017 and July 2024. For both groups, further data collected, if available, were surgical findings, histopathology, and postmortem examination findings. The control group was randomly selected from the non‐PE study population. Only the first CT examination meeting the inclusion criteria was selected for a control group case. Control group rabbits could have respiratory disease, but could not have evidence of PE on CT. The compliance with inclusion criteria was evaluated by a resident in small mammal medicine and surgery (A. A.) for clinical data and CT data by a diagnostic imaging resident (N. I.).

### 2.2 Clinical Data Analysis

Based on the patient's presenting signs, CT investigations were categorized into cardiac, dental, ear, endocrinological, metabolic, gastrointestinal, hepatic, musculoskeletal, neoplastic, ocular, neurological, upper respiratory tract, LRT, reproductive, integumentary, and urinary disease. Respiratory clinical signs were categorized into collapsed status, coughing, cyanosis, naso‐ocular discharge, dyspnea, neck extension, facial swelling, increased URT noise, open‐mouth‐breathing, reduced thoracic compliance, tachypnoea, and thoracic distension. Rabbits with no overt clinical respiratory signs were classified as having no respiratory signs. Secondary clinical signs of the respiratory disease were categorized as gut stasis, abdominal distension, reduced appetite, and inappetence. The clinical outcome was categorized into hospitalization for respiratory signs, oxygen dependency or other reasons, discharge from the hospital postdiagnosis, and death or euthanasia resulting from diagnosis. The survival time was recorded in days.

### 2.3 CT Scanning Procedure

All rabbits underwent a conscious CT examination, including the thorax, with a third‐generation 64‐row multidetector CT scanner (Somatom Definition AS, Siemens AG, Erlangen, Germany). Conscious restraint was facilitated using a 40 × 18 cm plexiglass tube restraining device (VetCatTrap, Universal Medical Systems, Solon, Ohio, USA) with the patient in sternal recumbency [15]. Flow‐by oxygen was provided throughout the scan, and visual stimulation was reduced by dimming light levels and placing a blanket over the restraint device. The floor of the device was covered with a towel to improve patient comfort, which minimized movement. The thoracic CT images were acquired with settings: 120 kV tube voltage, automated adaptive tube current, 0.33–1 s tube rotation time, 64 × 0.6 mm or 16 × 0.3 mm detector collimation configuration, 512 × 512 image matrix, and a collimator pitch of 1–1.5. Images were reconstructed with a high‐frequency kernel (Siemens proprietary name B70f or U80u) with a reconstructed slice width of 1 and 0.7 mm image interval. The image field‐of‐view was adapted to the animal's size, typically 18 to 20 cm in diameter. All images were stored in digital imaging and communications in medicine (DICOM) format on a local picture archiving and communication system (PACS).

### 2.4 Image Interpretation and Analysis

All images were retrieved from the PACS and analyzed by a diagnostic imaging resident (N. I.) in consensus with a boarded diagnostic imaging specialist (T. S.). The images were reviewed on a computer workstation (iMac 27‐inch, Apple Inc., Cupertino, CA, USA) with a calibrated LCD flatscreen monitor, using a dedicated, readily available open‐source DICOM viewer software (Horos, Purview, Annapolis MD, USA, version 3.3.6, 64‐bit; Nimble Co., LLC d/b/a Purview; https://www.horosproject.org) with a window width of 1400 Hounsfield units (HU) and a window level of −500 HU. The reviewers were blinded to presenting clinical signs, results from physical examination, outcome, and other relevant clinical data. They reviewed the study population for the presence of hypoattenuating lung areas (present/absent emphysematous lesions). Cases with emphysematous lesion(s) were included in the case group, and lesions were classified based on location in the right cranial, right medial, right caudal, right accessory, left cranial, and left caudal lung lobes [16]. Both segments of the left cranial lung lobe were considered as one lobe [16]. Each lung lobe was scored as nonemphysematous or having diffuse, peripheral, bullous, or mosaic pattern emphysema. Diffuse emphysema was defined as emphysema occupying the entire lung lobe. Peripheral emphysema was defined as being in the subpleural circumference of a lung lobe. Bullous emphysema was defined as a pulmonary gas pocket(s) surrounded by a thin rim of tissue, regardless of the location within the lobe. Mosaic pattern was defined as diffusely distributed areas of hypoattenuation lung between normally attenuating lung areas. Lung attenuation areas with positive HU values were excluded from evaluation. The pleural space was reviewed for the presence of free gas in the case and control groups and scored as having no pneumothorax, mild pneumothorax, or marked pneumothorax. Both groups were reviewed for any evidence of chest wall trauma, including fractures and dislocations of the ribs, sternum, and the thoracic vertebral column. In rabbits that underwent repeat CT examinations, the repeat interval between the first examination with evidence of emphysema and the last CT examination was recorded in days. Emphysema progression was scored based on the number of affected lung lobes and the emphysema volume. This was categorized as static (same number of lung lobes and <50% emphysema volume change), progressive (more lung lobes affected or ≥50% emphysema volume increase in the same number of affected lung lobes) or regressive (fewer lung lobes affected or ≥50% emphysema volume decrease in the same number of lung lobes). The emphysema volume was measured by manually contouring the emphysematous area on each CT slice using the pencil region of interest (ROI) tool and then applying the volume computing tool according to an established volumetry method [17]. A control group was extracted from the study population, randomly selecting rabbits without emphysematous lesions. Objective measurement of mean X‐ray attenuation in HU was performed and recorded for each lung lobe by placing a single 2.5 to 4 mm^2^ round to oval ROI into the center of the emphysema at the CT image with the maximum emphysema size for the case group, and into the center of the lobe for the control group, avoiding broncho‐vascular structures. For a nondiffuse emphysematous lesion in the case group, one additional ROI was placed in a nonemphysematous area in the same lobe.

### 2.5 Statistical Analysis

Statistical analyses were performed by one of the authors (D. C.), a diagnostic imaging resident with a Ph.D. in veterinary sciences and training in statistical analysis. Statistical analyses were performed using a commercial statistics software (Prism version 10.1.0 for macOS, GraphPad Software LLC, San Diego, CA, USA). The normality of the distribution was evaluated using the Shapiro–Wilk test. Descriptive statistics for age, sex, neuter status, weight, breed, environment, presenting complaint, and HU values of the lung lobes were calculated for both emphysematous and control groups. Clinical signs, PE location, medical and/or surgical treatment, case outcome, number of days of hospitalization, and survival time from diagnosis were also calculated in the emphysematous group. Depending on the distribution, data were reported as a percentage, mean ± standard deviation (SD), or median (range minimum to maximum). A chi‐square test was performed to assess statistical differences in the distribution of emphysema across lung lobes. To account for multiple comparisons, a Bonferroni‐corrected post hoc analysis was conducted to identify which lung lobes were significantly more affected. The adjusted significance level was calculated by dividing *α* = .05 by the number of pairwise comparisons, and results were interpreted accordingly. The prevalence of lower respiratory signs in the two groups (emphysematous vs. control) was compared using Fisher's exact test.

In the PE group, X‐ray attenuation in emphysematous lung lobes was compared with nonemphysematous areas within the same lobes using the Wilcoxon rank‐sum test. Additionally, X‐ray attenuation in emphysematous lobes from the PE group was compared with nonemphysematous lobes in the same group and to lung lobes in the control group using the Mann–Whitney test. The X‐ray attenuation of different types of emphysema was compared using the Kruskal–Wallis test, and results were further analyzed with Dunn's multiple comparison post hoc test.

For rabbits in the emphysema group with follow‐up CT examinations, descriptive statistics (median and range) for the number of newly affected lung lobes and volume change of emphysematous areas were calculated and expressed as a percentage for the first and last CT examinations. The results of repeat CT examinations were not included in the general analysis of the case group. In all analyses, *p* < .05 was considered statistically significant.

## 3 Results

In total, 724 CT studies of 527 rabbits were reviewed. Of these, 76 studies (10.5% of all studies) were positive for PE, involving 59 rabbits (11.2% of all rabbits). For the control group, 25 studies were randomly selected from the 648 PE‐negative cases.

### 3.1 Control Group

The control group of 25 rabbits (14 castrated males and 11 spayed females) had a median body weight of 2.5 kg (range 1.4–4.6 kg), with a mean (± SD) age of 7 years (±2 years). Represented breeds included: crossbreed (*n* = 10, 40%), unspecified breeds (*n* = 8, 32%), Netherland dwarf (*n* = 5, 20%), and English lop (*n* = 2, 8%). All rabbits were housed indoors. The reasons for presentation included: neurological disease (*n* = 6, 24%), persistent gastrointestinal stasis (*n* = 5, 20%), ear disease (*n* = 3, 12%), upper (*n* = 2, 8%) and lower (*n* = 3, 12%) respiratory disease, musculoskeletal disease (*n* = 2, 8%), ocular conditions (*n* = 1, 4%), skin disease (*n* = 1, 4%), urinary disease (*n* = 1, 4%), and dental disease (*n* = 1, 4%). The median X‐ray attenuation of the control group lung lobes was –652 HU (range –782 HU to –522 HU). None of the control group rabbits had a history of sustained thoracic trauma, and all of them were scored as having no pleural gas and no evidence of chest wall trauma on CT images.

### 3.2 Emphysema Group

**FIGURE 1:**
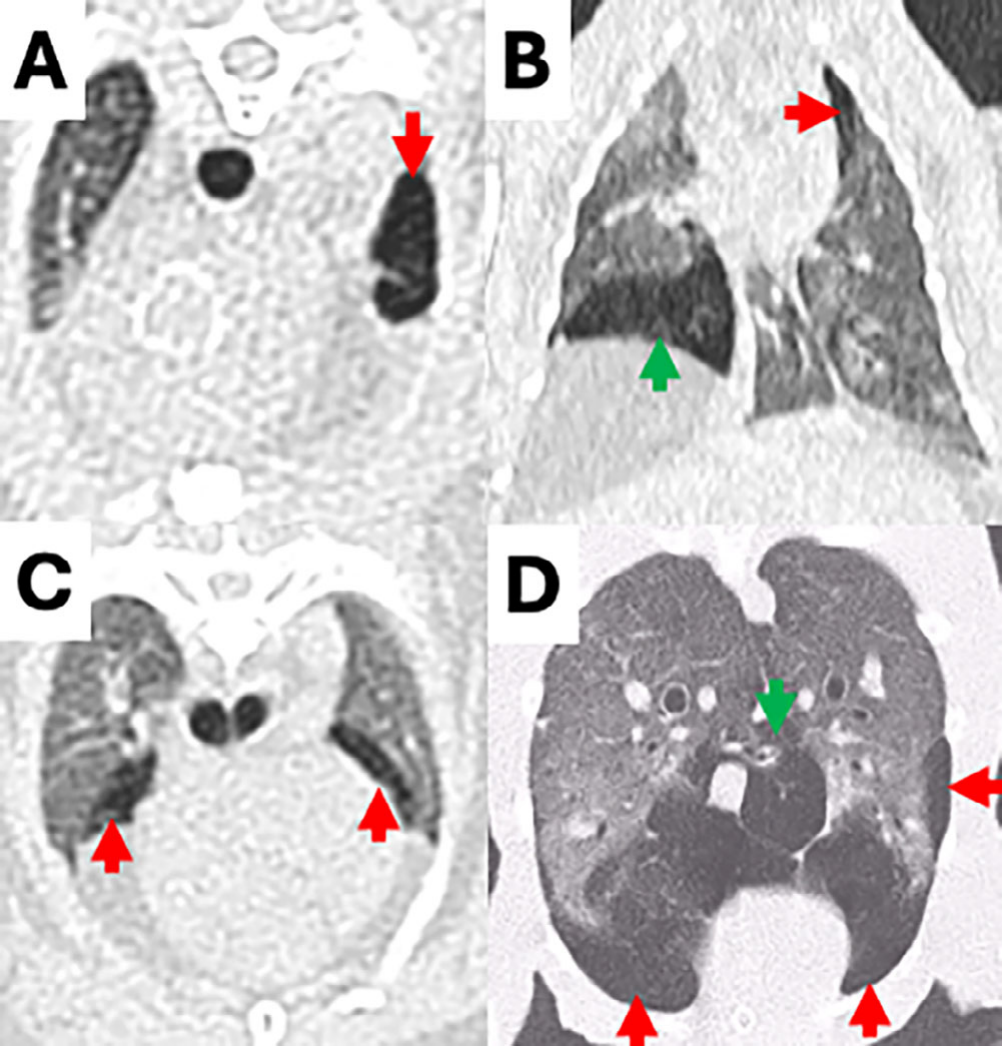
CT images of emphysematous hypoattenuating lung tissue in rabbits. (**A**) Unilobar in the left cranial lung lobe (red arrow), and multilobar in three different rabbits in (**B**) the left cranial (red arrow) and right middle (green arrow) lung lobes, (**C**) both cranial lung lobes (arrows) and (**D**) both caudal (red arrows) and accessory (green arrow) lung lobes.

**FIGURE 2:**
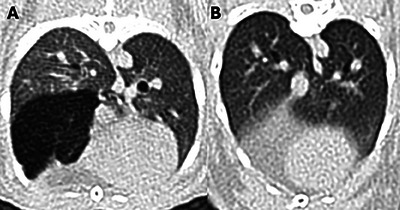
(**A**) Pulmonary emphysema in a rabbit with a hypoattenuating right caudal lung lobe with a mean attenuation of −915 HU, compared with (**B**) the same lung lobe in a normal control rabbit with a measured mean attenuation of −700 HU. Emphysematous lung areas are visibly and measurably hypoattenuating compared with normal lungs.

The emphysema group included 59 rabbits (31 castrated males, 27 spayed females, and 1 intact female) with a total of 133 emphysematous and 221 nonemphysematous lung lobes. The median body weight was 2.7 kg (range 1–6.9 kg), and the mean (± SD) age was 9 (±4) years (range 5–13 years). Represented breeds included: crossbreed (*n* = 26, 44%), no specified breed (*n* = 13, 22%), Flemish giant (*n* = 6, 10.2%), Netherland dwarf (*n* = 4, 6.8%), Rex (*n* = 4, 6.8%), English lop (*n* = 3, 5.1%), and French lop (*n* = 3, 5.1%). Forty‐three rabbits (72.89%) were housed indoors, nine (15.25%) were housed outdoors, and seven (11.86%) had a combined indoor‐outdoor environment. Presenting reasons included: upper respiratory disease (*n* = 12, 20.3%), persisting gastrointestinal stasis (*n* = 9, 15.2%), lower respiratory disease (*n* = 8, 13.5%), ear disease (*n* = 7, 11.9%), ocular conditions (*n* = 4, 6.8%), dental disease (*n* = 4, 6.8%), musculoskeletal conditions (*n* = 4, 6.8%), neurological disease (*n* = 3, 5.1%), cardiac disease (*n* = 3, 5.1%), integumentary conditions (*n* = 2, 3.4%), hepatic disease (*n* = 1, 1.7%), neoplasia (*n* = 1, 1.7%), and urinary disease (*n* = 1, 1.7%). None of the case group rabbits had a history of thoracic trauma. No rabbit presented in collapsed status, exhibited facial swelling, abdominal, or thoracic distension. Thirteen rabbits (22%) presented in gut stasis, while two other rabbits (3.4%) showed solely signs of gut stasis, without any respiratory signs. Twenty‐six rabbits (44.1%) were asymptomatic and underwent CT investigation for a nonrespiratory‐related issue. Clinical signs are summarized in Table 1. For 56 rabbits (94.9%), no information regarding exposure to environmental irritants was available. One rabbit (1.7%) was exposed to tobacco smoke, while two others (3.4%) had candles in the same room as their enclosure. Considering the 59 rabbits at presentation, emphysema was confined to a single lobe in 30 cases (50.8%), while 29 rabbits (49.2%) exhibited multilobar involvement (Figure 1). Of the 133 lung lobes initially affected, the most commonly involved were the left cranial (*n* = 40, 30.1%) and right cranial (*n* = 39, 29.3%) lobes, followed by the right middle (*n* = 15, 11.3%), left caudal (*n* = 14, 10.5%), right caudal (*n* = 13, 9.8%), and right accessory (*n* = 12, 9%) lobes. In the PE group, the median X‐ray attenuation of emphysematous lung lobes was −905 HU (range −1000 to −729 HU) in the affected lung areas and −667 HU (range −840 to −395 HU) in the unaffected areas. In nonemphysematous lung lobes of the PE group, the median X‐ray attenuation was −662 HU (range −863 to −730 HU) (Figure 2).

**TABLE 1:** Respiratory and generalized clinical signs observed in rabbits with pulmonary emphysema (*n* = 59).

Clinical signs	Number of rabbits (*n* = 59)	Percentage (%)
No signs	26	44.1
Respiratory signs	33	55.9
Dyspnea	13	22
Increased URT noise	10	17
Naso‐ocular discharge	6	10.2
Cyanosis	6	10.2
Facial swelling	6	10.2
Tachypnoea	5	8.5
Neck extension	3	5.1
Open mouth breathing	1	1.7
Reduced thoracic compliance	1	1.7
Coughing	1	1.7
Generalized signs		
Gut stasis	13	22
Reduced appetite	4	6.8
Inappetence	1	1.7

*Note*: The percentages are not cumulative since more than one clinical sign was noted in several rabbits.

**FIGURE 3:**
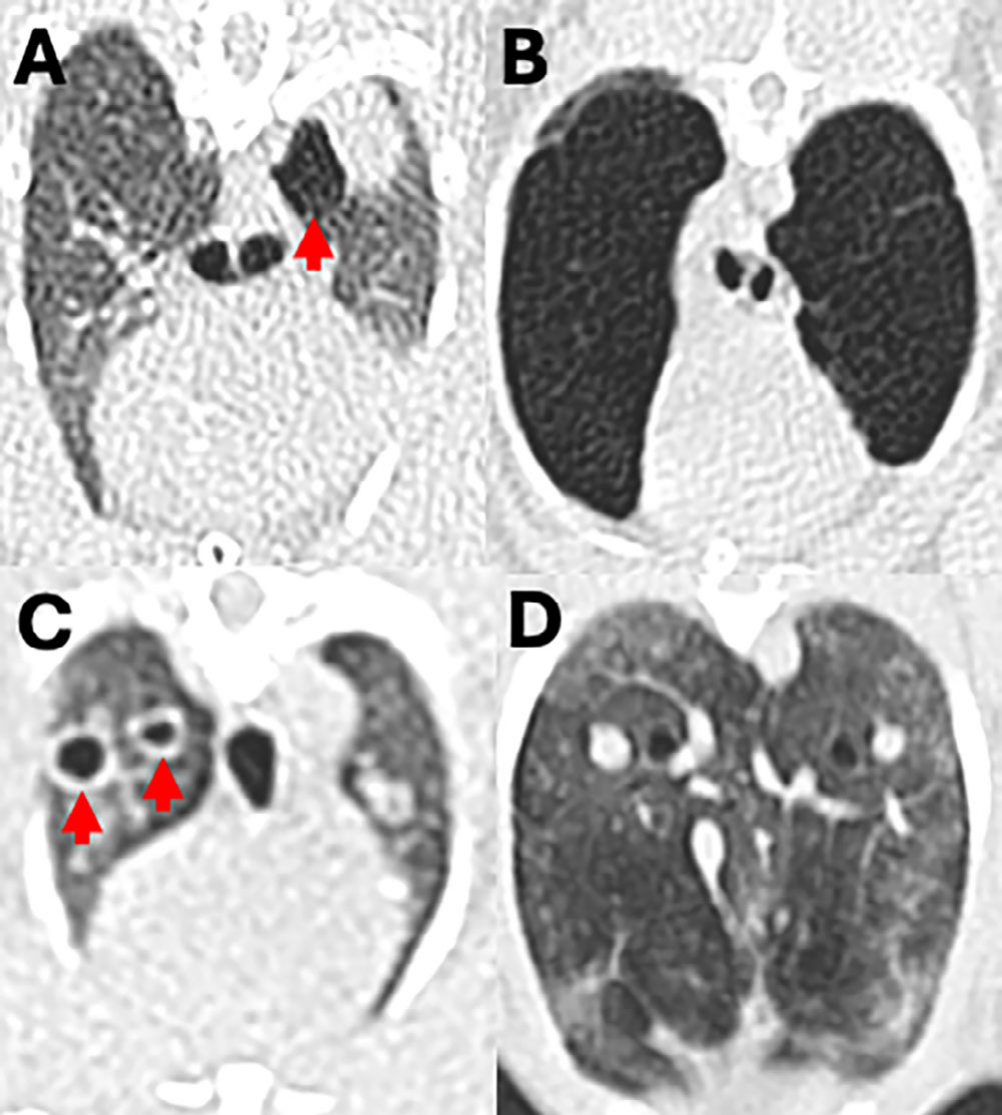
Different emphysema patterns: (**A)** peripheral, (**B)** diffuse, (**C)** bullous, and (**D)** mosaic pattern. Arrows point out areas of localized emphysema.

**FIGURE 4:**
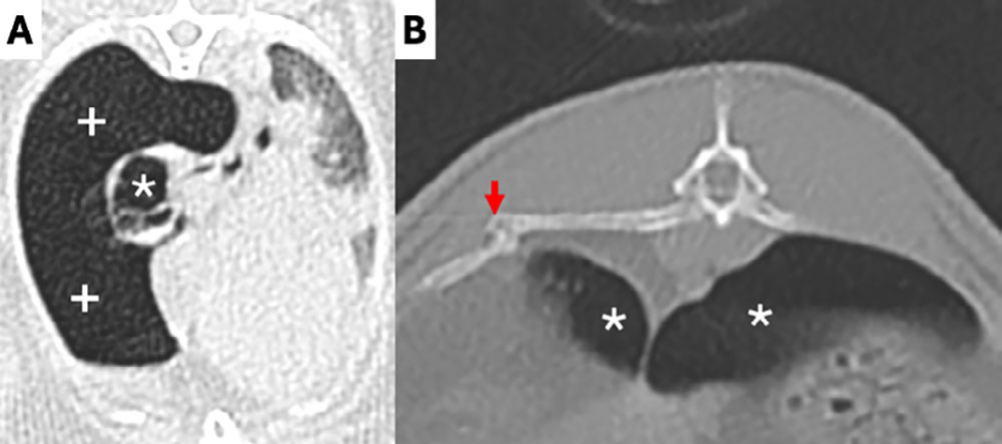
(**A**) CT image of a 9‐year‐old rabbit with acute severe dyspnea without a trauma history. There is bullous emphysema (*) in the otherwise collapsed right cranial lung lobe and a large amount of free gas in the right pleural cavity (+), consistent with spontaneous tension pneumothorax. (**B**) Fracture (arrow) of the proximal portion of the 11th right rib in a 6‐year‐old rabbit with chronic dyspnea without a trauma history. There was marked diffuse emphysema (*) in all lobes. This is most likely a pathologic fracture secondary to chronic increased respiratory effort.

The emphysema pattern was peripheral in 79, diffuse in 41, bullous in 8, and mosaic in 5 lung lobes (Figure 3). The median X‐ray attenuation was −850 HU for diffuse (range −968 to −754 HU), ‐922 HU for peripheral (range −1000 to −729 HU), −952 HU for bullous (range −1000 to −859 HU), and −862 HU for mosaic (range −914 HU to −807 HU) emphysema patterns. There were 57 case group rabbits with no pneumothorax, while 2 of the rabbits with bullous emphysema had marked unilateral right pneumothorax (Figure 4A). There were three rabbits with each a single rib fracture in the mid‐portion of the 6th, and proximal portion of the 11th and 12th right rib with rounded margins, respectively, but no rabbit with evidence of sternal or vertebral fractures or dislocations (Figure 4B). There were 15 rabbits in the case group with at least one repeat CT examination. The median interval duration between the first CT examination with evidence of PE and the last CT examination was 369 days (range 28 to 1238 days).

**FIGURE 5:**
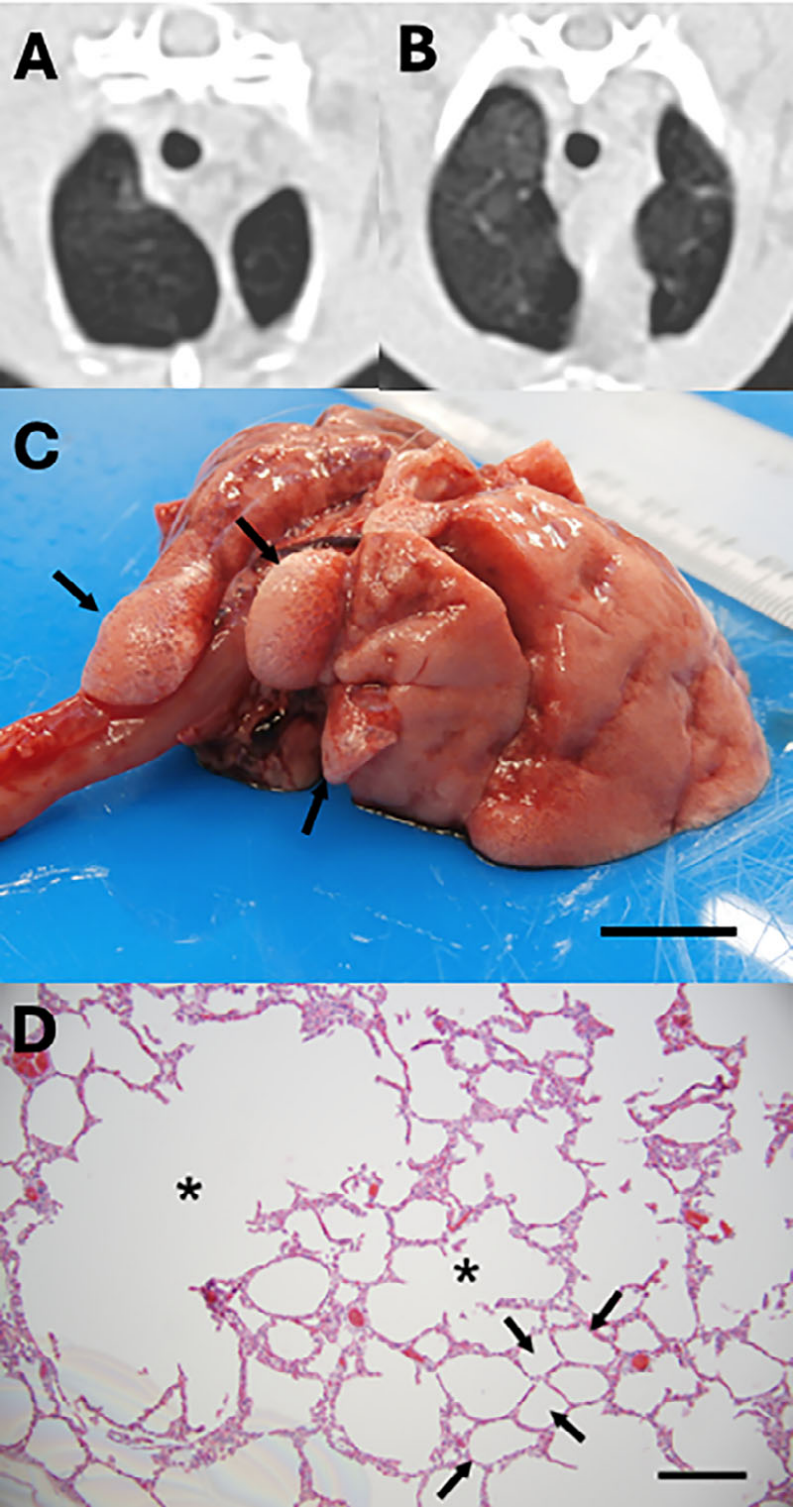
(**A, B**) Consecutive transverse CT images with hypoattenuating emphysematous cranial lung lobes in a 2.5‐year‐old rabbit that was euthanized due to progressive dyspnea. (**C**) At postmortem examination, there was moderate to severe emphysema in all lung lobes, which was severe (arrows), mostly in the cranial lobes (scale bar = 1.5 cm). (**D**) Microscopically, there was multifocal to coalescing alveolar expansion and coalescence, consistent with emphysema (scale bar = 150 µm) *Emphysematous alveoli, arrows: normal‐sized alveoli.

Depending on the individual case, the medical management involved a range of treatments, and many rabbits received more than one treatment (Table 2). Three years after the conclusion of image data collection, of the 59 rabbits of the emphysema group, 27 (45.8%) were still alive, 20 (33.9%) were deceased within one month from the diagnosis and 6 (10.2%) deceased within the following twelve months, while the remaining 6 (10.2%) died between 14 months to 3 years from diagnosis. In one rabbit (1.7%), a complete postmortem examination was performed 30 days post‐CT, and a histopathological examination revealed multifocal to coalescing alveolar expansion and coalescence, consistent with severe to moderate bilateral PE (Figure 5).

**TABLE 2:** Overview of the treatment of rabbits with pulmonary emphysema (*n* = 59).

Treatment	No. of rabbits (*n* = 59)
Antibiotics	
Enrofloxacin	10
Doxycycline	1
Trimethoprim/Sulfamethoxazole	1
Metronidazole	1
Anti‐inflammatories	
Meloxicam	13
Opioid analgesia	
Injectable buprenorphine	5
Bronchodilators	
Injectable (Terbutaline)	2
Inhalant (Salbutamol)	2
Mucolytics	
Bromhexine (per os)	7
Nebulization therapy	
F10 Antiseptic solution diluted in saline (1:250)	13
Steroid therapy	
Dexamethasone (injectable)	1
Prednisolone (per os)	3
Oxygen provision	6

### 3.3 Statistical Analysis

**FIGURE 6:**
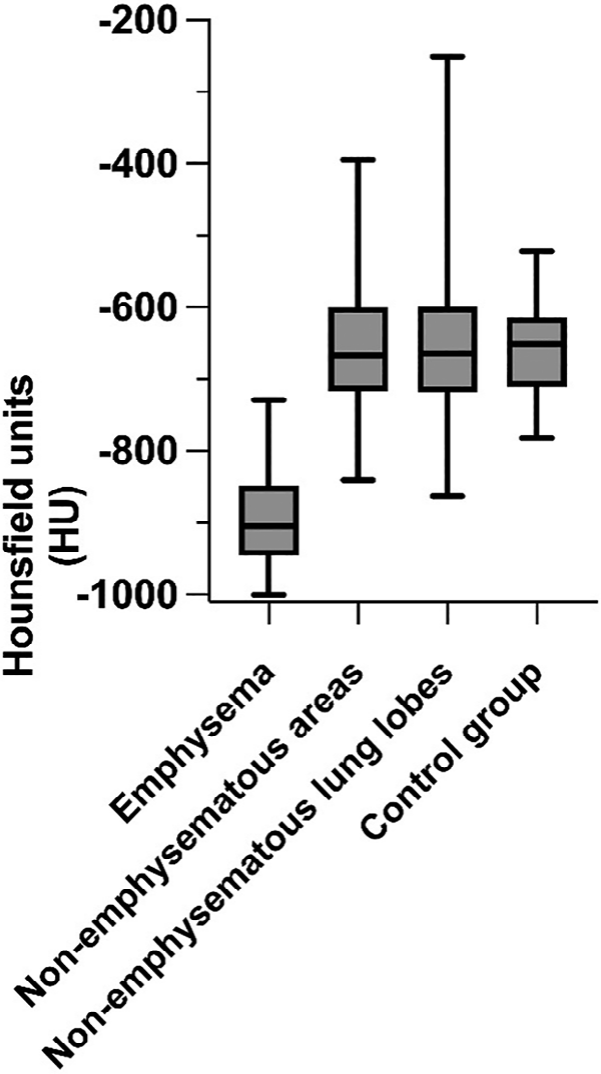
Box and whisker plot showing significant differences in pulmonary X‐ray attenuation in Hounsfield units (HU) between emphysematous lung lobes, nonemphysematous areas of the affected lung lobes of the emphysema group, nonemphysematous lung lobes, and lung lobes of the control group. The line within the box represents the median, the upper and lower sides of the box are the lower and upper quartiles, and the two extreme horizontal lines represent the minimum and maximum values. Emphysematous lung areas are significantly less attenuating than nonemphysematous lung areas or lobes.

**FIGURE 7:**
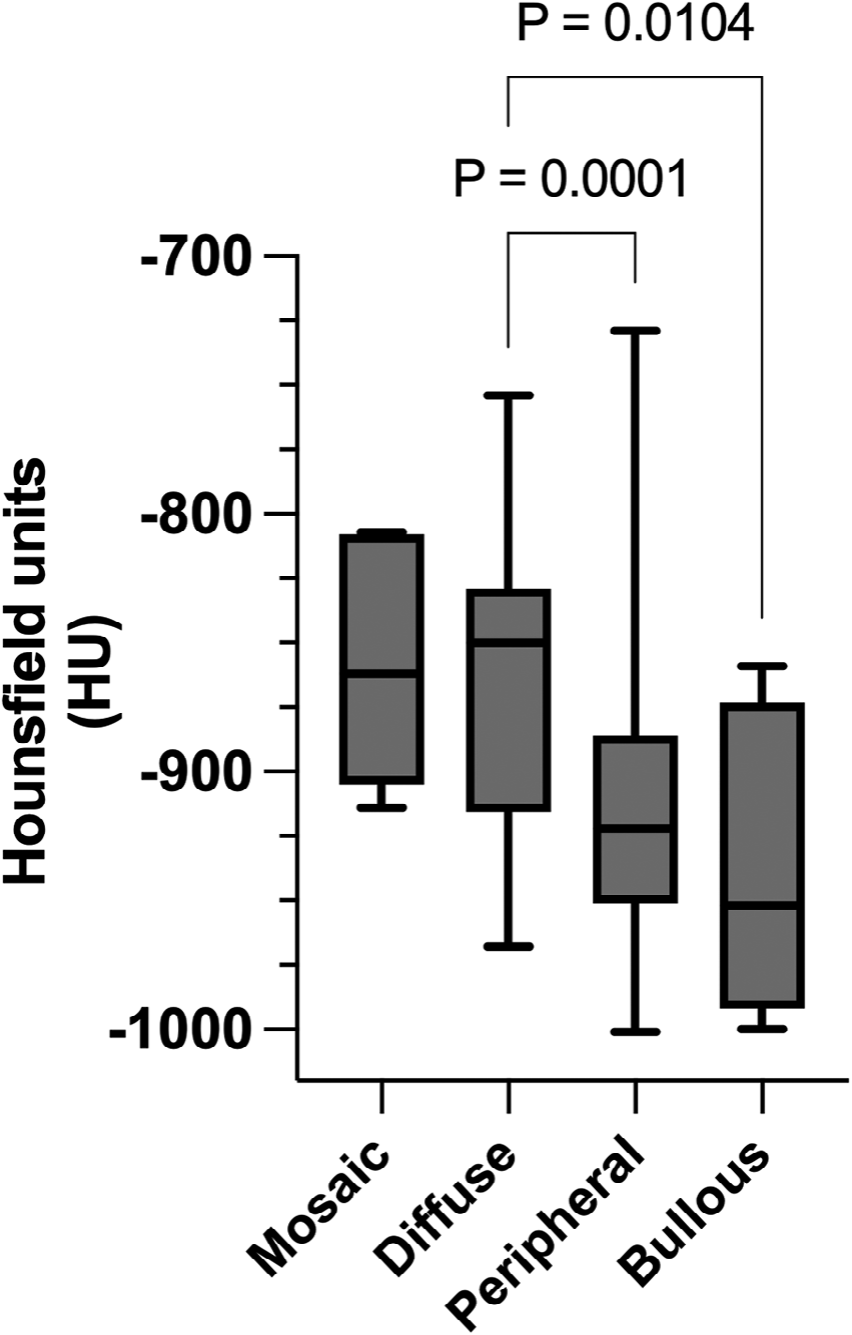
Box and whisker plot comparing X‐ray attenuation in HU in emphysematous lung lobes with different patterns. The line within the box represents the median, the upper and lower sides of the box are the lower and upper quartiles, and the two extreme horizontal lines represent the minimum and maximum values. Peripheral and bullous emphysema lobes are significantly less attenuating than diffuse emphysema lobes. The mosaic pattern group was too small to apply statistical analysis.

**FIGURE 8:**
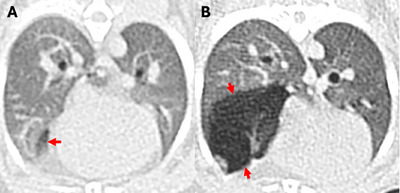
Example of a rabbit with progressive pulmonary emphysema. (**A**) At 2 years of age, there was mild peripheral emphysema in the right caudal lung lobe (arrow). (**B**) On repeat examination 4 years later, there was marked progression of emphysema in the same lobe (arrows).

The chi‐square test revealed a statistically significant difference in the distribution of emphysema across lung lobes (χ^2^ = 43.64, df = 5, *p* < .0001). The post hoc Bonferroni analysis showed that the left cranial lobe was significantly more affected compared with the right middle, left caudal, right caudal, and right accessory lobes (*p* < .01), while no significant difference was found between the left and right cranial lobes (*p* = 1.000). Fisher's exact test did not find a statistically significant difference in the incidence of the LRT signs in the emphysematous group compared with the control group (*p* = .18). The Wilcoxon rank‐sum test demonstrated a statistically significant difference (*p* < .0001) in X‐ray attenuation between emphysematous (*n* = 133) and nonemphysematous areas of affected lung lobes (*n* = 91). The Mann–Whitney test also demonstrated a statistically significant difference between emphysematous areas of emphysematous lung lobes and nonemphysematous lung lobes (*n* = 221) of the emphysema group, and between the emphysematous areas of emphysematous lung lobes and the lung lobes of the control group (*n* = 150); (all *p* < .0001) (Figure 6). The Kruskal–Wallis test and Dunn's multiple comparison post hoc tests revealed a statistically significant difference in X‐ray attenuation between lung lobes with diffuse emphysema and those with peripheral and bullous emphysema (*p* = .0001 and *p* = .01, respectively, Figure 7). Of the 15 rabbits with repeat CT examination, 12 (80%) were classified as having progressive disease (Figure 8) and 3 (20%) as having static disease. Among the 12 rabbits with progressive disease, 8 (53.33%) had involvement of at least one additional lung lobe (median = 1, range = 1–5), while the remaining 4 (26.67%) showed no additional lung lobe involvement but exhibited a ≥50% increase in emphysema volume (median increase = 174.5%, range = 75%–303%) on the repeat CT examination. Of the 3 (20%) rabbits classified as having static disease, one (6.67%) had emphysema in all lung lobes, with most of the lung parenchyma already affected at the first CT exam. Of the 59 rabbits in the emphysema group, 4 (6.8%) were hospitalized due to the observed respiratory signs, while 2 (3.4%) were hospitalized due to being oxygen‐dependent. Thirteen rabbits (22%) were euthanized due to marked or refractory respiratory disease, and none underwent surgical treatment for PE. Of these rabbits, 10 cases had multilobar emphysema and 7 had lower X‐ray attenuation values than the observed median (−905 HU). Among the thirteen euthanized rabbits, the emphysema pattern was diffuse in 5, peripheral in 4, bullous in 3, and mosaic in 1. The two rabbits with pneumothorax belonged in the euthanized group. Two other rabbits have evidence of presumed pulmonary neoplasia.

## 4 Discussion

This is the first large‐scale study investigating PE in domestic rabbits. Our hypothesis that PE is a common CT finding was confirmed, with 11.2% of the study population being affected. Anatomical (e.g., small‐sized thorax) and physiological (e.g., obligate nasal breathers) features of rabbits render them prone to respiratory disease [18]. Across species, causes of PE have been associated with chronic airway inflammation with subsequent bronchial constriction, shortening of the cilia of the respiratory epithelium, bronchial wall collapse, diaphragmatic hernia, pulmonary neoplasia, exposure to environmental irritants, such as dust or mould in hay or straw, and repeated exposure to tobacco smoke [2, 19, 20]. PE was identified histopathologically in 14 out of 17 animals (14 dogs and 3 cats) in one study and one rabbit in our investigations [2]. The incidence of PE in rabbits appears to be higher, reaching approximately 83% in one study in experimental rabbits, in which PE was not experimentally induced [14]. This could be related to the housing conditions of rabbits. Domestic rabbits live at a lower level than the other species and are repeatedly exposed to potential air‐borne irritants such as dust, which was shown to cause allergic airway inflammation in a study involving 38 rabbits [11]. One rabbit in our study with PE lived in an environment with indoor accumulation of tobacco smoke, which has been linked with PE in rabbits in one experimental study [12]. The high median age of the rabbits with PE in our study (9 years) suggests that emphysema is linked to low‐impact, noxious effects leading to chronic changes. A potential consequence of bullous PE is bulla rupture, leading to spontaneous pneumothorax, which has been reported in cats and dogs [2, 21]. Pneumothorax is rarely reported in rabbits, with information being limited to one case report and one case series [8, 9]. Pneumothorax is usually associated with dyspnea, which was the case in both rabbits affected by pneumothorax in our study [20]. Both rabbits had bullous emphysema but no trauma history. Interestingly, in both of our cases with pneumothorax, and 4 out of 5 reported cases, the pneumothorax was unilateral (3 left and 3 right), suggesting that rabbits, unlike dogs and cats, do not possess intrapleural communications [8, 9]. The rib fractures in the three rabbits were chronic and could represent fatigue fractures related to chronic increased respiratory efforts. These fractures were located at the margin of the serratus dorsalis cranialis and caudalis muscles, which are one of the few expiratory muscles. With hypertrophy of these muscles, the shearing forces with the remainder of the chest wall promote rib fractures at this location [22]. The presence of pneumothorax or rib fractures in imaging studies of rabbits should alert clinicians to potential PE since, due to their housing conditions, rabbits are less prone to thoracic trauma compared with other domestic animals. However, specific housing arrangements, for example, the presence of wired room dividers, may favor thoracic trauma due to entrapment after jumping over them. Our hypothesis, that PE correlates with clinical signs of LRT disease, was not confirmed. Almost half (*n* = 26, 44.1%) of the rabbits with PE in this study were asymptomatic. In an experimental study, this percentage was even higher (83%), likely related to the short life span of experimental animals [14]. The absence of clinical signs is also anticipated to a degree due to the prey behaviour of rabbits. As rabbits encounter novel environments (e.g., the hospital setting), they can become hypervigilant of potential threats, which inhibits behavioural changes caused by disease and pain [23]. A slight majority of cases (55.9%) had clinical respiratory disease, but this was not significantly different from the control group. Dyspnea and increased respiratory effort are clinical signs seen in humans, horses, cats, and dogs with PE and can be expected in rabbits with advanced PE [1, 2, 8, 18]. Open‐mouth breathing and cyanotic mucous membranes indicate severe disease, and rabbits presenting with these clinical signs carry a grave prognosis. This is consistent with our study in which a small proportion of rabbits with these clinical symptoms (*n* = 6, 10.5%) were severely ill and were euthanized on welfare grounds.

Chronic lower airway disease in cats and horses, which can lead to diffuse emphysema, is more commonly seen in the caudal lobes [2, 24]. In our study, the cranial lung lobes were significantly more affected than the other lobes. The reason for the predilection of the distribution of PE in these lobes is unknown, but could be related to the pulmonary anatomical characteristics in the rabbit. These include the small and narrow shape and mobility of the cranial lobes, minimal interlobular connective tissue, short bronchioles, reduced lumen diameter, and cartilage hypoplasia in cases with congenital PE [25, 26]. These features may increase the likelihood of pulmonary tissue retraction and alveolar collapse, leading to inflation and the development of PE.

Our hypothesis, that the X‐ray attenuation of the emphysematous lung lobes would be reduced, was confirmed, and this was the case both compared with nonemphysematous areas in the same lobe, nonemphysematous lobes in the same animal, and lungs of control group animals. Air does not attenuate X‐rays, whereas soft tissue does. In emphysema, the alveolar lining of the lungs is destroyed, leading to large pockets of air in the lungs. The median X‐ray attenuation of emphysematous lungs in this study (−905 HU) was slightly higher than what has been reported in humans (< −950 HU), dogs, and cats (mean −949 HU) [2, 27]. This may be related to the smaller size of rabbit lungs, not allowing measurement of completely tissue‐free lung areas. In our study, X‐ray attenuation of nonemphysematous lungs in the case (median −667 HU) and control group (median −652 HU) was slightly lower than in other studies of normal lungs of sedated (mean −610 HU right, −634 HU left lung) or anaesthetized rabbits (mean −549 HU right, −583 left lung) [24, 28]. This is consistent with the conscious status and natural sternal recumbency of the rabbits in our study, which minimizes the tendency for lung atelectasis. For anaesthetic induction, animals are often placed in lateral recumbency, promoting lung atelectasis. Another factor could also be breed‐specific variations in lung density since New Zealand White rabbits used in both previous studies have a higher body weight (4–5 kg) than the median body weight of the rabbits in our study (2.7 kg) [25, 28].

Our hypothesis that PE would be progressive was confirmed, with 80% of cases showing signs of emphysema progression. Emphysema is a progressive, irreversible disease. Our study demonstrates that CT is an appropriate tool for monitoring the disease progression in rabbits. Given that rabbits often do not have LRT signs in the early phase of the disease but can have severe dyspnea at end‐stage disease, CT might be a valuable tool for prognosis and monitoring.

Limitations of this study include the lack of histopathological examination to exclude microscopic emphysema in the subjects included in the control group. As demonstrated in our study, pulmonary emphysema was an incidental finding in a significant proportion of cases and was not associated with clinical signs. Consequently, we expect that even subjects with microscopic emphysema would likely have a similar clinical course (i.e., asymptomatic). From this perspective, a complete histopathological examination of all subjects in the control group, not only those with respiratory disease, would be necessary to confidently rule out microscopic emphysema. However, we believe that obtaining a necropsy examination for every subject in such a large control group would be extremely challenging, even in a prospective study, and quite unrealistic given the practical and logistical difficulties of performing necropsies on a sample of this size. On the other hand, using clinical cases allowed for the investigation of the prevalence, distribution, and progression of pulmonary emphysema, which would differ from the findings of a postmortem study. Our control group intentionally contained rabbits with respiratory disease since excluding these subjects would have introduced selection bias, potentially skewing the data and compromising the study's representativeness. Excluding subjects with respiratory signs from the control group would have resulted in an unbalanced comparison, as the case group includes subjects with respiratory signs, while the control group would not. This imbalance could have artificially strengthened the perceived association between respiratory signs and emphysema, since respiratory signs would have been exclusively present in subjects with emphysema. Although we acknowledge that the control group may include subjects with pulmonary emphysema that is not evident on CT, this possibility is quite remote, given the high sensitivity of the modality in detecting such alterations. On the other hand, the exclusion of symptomatic subjects, in our opinion, would have had a greater impact for the reasons mentioned above.

In conclusion, PE is a relatively common condition in older domestic rabbits, which may be asymptomatic in the early stage of the disease but can lead to dyspnea at advanced stages. On CT, PE manifests as hypoattenuating areas of the pulmonary parenchyma with significantly lower X‐ray attenuation compared with nonemphysematous lungs or lung areas. Rare consequences or chronic advanced PE include rib fractures and pneumothorax. PE is a chronic, progressive disease. PE should be included as a differential diagnosis in rabbits with respiratory signs, and the progressive nature of the disease should be considered when detected in asymptomatic rabbits.

## Author Contributions

**Category 1**

(a) Conception and Design: Schwarz, Athinodorou, Richardson, Israeliantz.
(b) Acquisition of Data: Athinodorou, Israeliantz.
(c) Analysis and Interpretation of Data: Athinodorou, Costanza, Schwarz, Israeliantz, Richardson, del Pozo.

**Category 2**

(a) Drafting the Article: Athinodorou.
(b) Revising the Article for Intellectual Content: Schwarz, Costanza, Richardson, del Pozo, Israeliantz.

**Category 3**

(a) Final Approval of the Completed Article: Athinodorou, Schwarz, Richardson, Costanza, del Pozo, Israeliantz.

## Conflicts of Interest

The authors declare no conflicts of interest.

## Publication disclosure

Part of the results from this study were presented as oral communication at the ECVDI Congress, Edinburgh, United Kingdom, September 14–17, 2022.

## Data Accessibility Disclosure

Supporting data are available from the corresponding author upon reasonable request.

## Reporting Checklist Disclosure

Authors followed the Strobe‐VET network guideline disclosure.

## References

[1] M. Takahashi , “Imaging of Pulmonary Emphysema: A Pictorial Review,” International Journal of Chronic Obstructive Pulmonary Disease 3 (2008): 193–204. 18686729 10.2147/copd.s2639 PMC2629965

[2] H. Warwick , J. Guillem , D. Batchelor , et al., “Imaging Findings in 14 Dogs and 3 Cats With Lobar Emphysema,” Journal of Veterinary Internal Medicine 35, no. 4 (2021): 1935–1942. 34145623 10.1111/jvim.16183 PMC8295672

[3] C. Hong , H. Deng , M. Li , et al., “Gene Expression Profiling Reveals Differential Patterns Between Microcystic Congenital Cystic Adenomatoid Malformation and Congenital Lobar Emphysema,” Early Human Development 128 (2019): 77–80. 30583279 10.1016/j.earlhumdev.2018.12.014

[4] S. Del Magno , S. Zanardi , A. Foglia , et al., “Congenital Lobar Emphysema in a Kitten With Concomitant Hiatal Hernia and Nutritional Secondary Hyperparathyroidism,” Journal of the American Animal Hospital Association 58, no. 3 (2022): 141–145. 35576398 10.5326/JAAHA-MS-7151

[5] H. J. Han and J. H. Kim , “Concurrent Pulmonary Hypoplasia and Congenital Lobar Emphysema in a Young Dog With Tension Pneumothorax: A Rare Congenital Pulmonary Anomaly,” Acta Veterinaria Scandinavica 61, no. 1 (2019): 37. 31349870 10.1186/s13028-019-0472-2 PMC6659239

[6] P. Rajkumar , K. Pattabi , S. Vadivoo , et al., “A Cross‐sectional Study on Prevalence of Chronic Obstructive Pulmonary Disease (COPD) in India: Rationale and Methods,” BMJ Open 7, no. 5 (2017): e015211. 10.1136/bmjopen-2016-015211 PMC5729985 28554925

[7] D. Marinkovic , S. Aleksic‐Kovacevic , and P. Plamenac , “Cellular Basis of Chronic Obstructive Pulmonary Disease in Horses,” International Review of Cytology 257 (2007): 213–247. 17280899 10.1016/S0074-7696(07)57006-3

[8] F. Guillerit , L. Gros , C. Touzet , P. M. Delattre , M. Huynh , and A. Girard‐Luc , “Spontaneous Pneumothorax in Four Pet Rabbits (2017–2022),” Journal of Exotic Pet Medicine 45 (2023): 30–37.

[9] G. R. Browning , J. W. Carpenter , K. Tucker‐Mohl , M. Drozd , and A. G. Cino‐Ozuna , “What Is Your Diagnosis?,” Journal of the American Veterinary Medical Association 256, no. 8 (2020): 873–877. 32223713 10.2460/javma.256.8.873

[10] K. Williams and J. Roman , “Studying human respiratory Disease in Animals—role of Induced and Naturally Occurring Models,” Journal of Pathology 238, no. 2 (2016): 220–232. 26467890 10.1002/path.4658

[11] A. Divaret‐Chauveau , L. Foucaud , B. Demoulin , et al., “Early Exposure to Farm Dust in an Allergic Airway Inflammation Rabbit Model: Does It Affect Bronchial and Cough Hyperresponsiveness?,” PLoS ONE 18, no. 1 (2023): e0279498. 36706084 10.1371/journal.pone.0279498 PMC9882901

[12] F. Fidan , M. Unlu , M. Sezer , O. Sahin , C. Tokyol , and H. Esme , “Acute Effects of Environmental Tobacco Smoke and Dried Dung Smoke on Lung Histopathology in Rabbits,” Pathology 38, no. 1 (2006): 53–57. 16571591 10.1080/00313020500459615

[13] H. T. Strawbridge , “Chronic Pulmonary Emphysema (An experimental study) II. Spontaneous Pulmonary Emphysema in Rabbits,” American Journal of Pathology 37, no. 3 (1960): 309–331. 13835161 PMC1942307

[14] T. K. Cooper , J. W. Griffith , Z. C. Chroneos , J. M. Izer , L. B. Willing , and X. Peng , “Spontaneous Lung Lesions in Aging Laboratory Rabbits (*Oryctolagus cuniculus*),” Veterinary Pathology 54, no. 1 (2017): 178–187. 27507806 10.1177/0300985816658102

[15] C. R. Oliveira , F. N. Ranallo , G. J. Pijanowski , et al., “The VetMousetrap: A Device for Computed Tomographic Imaging of the Thorax of Awake Cats,” Veterinary Radiology & Ultrasound: The Official Journal of the American College of Veterinary Radiology and the International Veterinary Radiology Association 52, no. 1 (2011): 41–52. 21322386

[16] P. Popesko , V. Ratjova , and J. Horak , A Colour Atlas of Small Laboratory Animals. Volume One: Rabbit. Guinea Pig (Wolfe, 1992): 72–73.

[17] N. Israeliantz , J. Lodzinska , G. Woods , J. Pontes , M. Parys , and T. Schwarz , “A Simplified CT‐Volumetry Method for the Canine Liver,” Veterinary Radiology & Ultrasound: The Official Journal of the American College of Veterinary Radiology and the International Veterinary Radiology Association 63, no. 1 (2022): 47–53. 34806252 10.1111/vru.13018

[18] A. M. Lennox and E. Mancinelli . Chapter 15: Respiratory Disease. In: K. E. Quesenberry , C. J. Orcutt , C. Mans , J. W. Carpenter , eds. Ferrets, Rabbits, and Rodents Clinical Medicine and Surgery. (Elsevier, 2020): 188–200.

[19] A. K. Barton and H. Gehlen , “Pulmonary Remodelling in Equine Asthma: What Do We Know About Mediators of Inflammation in the Horse?,” Mediators Inlamm 1 (2016): 5693205. 10.1155/2016/5693205 PMC5174180 28053371

[20] M. Hikichi , K. Mizumura , S. Maruoka , and Y. Gon , “Pathogenesis of Chronic Obstructive Pulmonary Disease (COPD) Induced by Cigarette Smoke,” Journal of Thoracic Disease 11, no. 17 (2019): S2119–s2140. 10.21037/jtd.2019.10.43 PMC6831915 31737341

[21] C. Gilday , A. Odunayo , and A.‐M. Hespel , “Spontaneous Pneumothorax: Pathophysiology, Clinical Presentation and Diagnosis,” Topics in Companion Animal Medicine 45 (2021): 100563. 34303864 10.1016/j.tcam.2021.100563

[22] E. M. Hardie , O. R. Ill , J. N. Clary , et al., “Abnormalities of the Thoracic Bellows: Fractures of the Ribs and Hiatal Hernia,” Journal of Veterinary Internal Medicine 12 (1998): 279–287. 9686388 10.1111/j.1939-1676.1998.tb02123.x

[23] E. A. McBride , “Small Prey Specie's Behaviour and Welfare: Implications for Veterinary Professionals,” JSAP 58, no. 8 (2017): 423–436. 10.1111/jsap.12681 28513850

[24] P. M. Dixon , D. I. Railton , and B. C. McGorum , “Equine Pulmonary Disease: A Case Control Study of 300 Referred Cases. Part 3: Ancillary Diagnostic Findings,” Equine Veterinary Journal 27, no. 6 (1995): 428–435. 8565939 10.1111/j.2042-3306.1995.tb04423.x

[25] D. Müllhaupt , S. Wegner , P. Kircher , N. Pfammatter , J.‐M. Hatt , and S. Ohlerth , “Computed Tomography of the Thorax in Rabbits: A Prospective Study in Ten Clinically Healthy New Zealand White Rabbits,” Acta Veterinaria Scandinavica 59 (2017): 72. 29065887 10.1186/s13028-017-0340-x PMC5655941

[26] J. L. Peake and K. E. Pinkerton . Chapter 3 – Gross and Subgross Anatomy of Lungs, Pleura, Connective Tissue Septa, Distal Airways, and Structural Units. In: R. A. Parent , ed. Comparative Biology of the Normal Lung Second Edition. (Elsevier, 2015): 21–31.

[27] B. M. Smith , J. H. M. Austin , and J. D. Newell Jr , et al., “Pulmonary Emphysema Subtypes on Computed Tomography: The MESA COPD Study,” American Journal of Medicine 127, no. 1 (2014): 94.e7–23. 10.1016/j.amjmed.2013.09.020 PMC3882898 24384106

[28] R. F. J. Sarjo , I. O. Tomé , F. C. Silva , and M. M. D. Ginja , “Quantitative CT Evaluation of Lung Volume and Density in Sedated and Anaesthetised Rabbits (abstract) IVRA EVDI Joint Scientific Conference Dublin, Ireland, June 18–23,2023,” Veterinary Radiology & Ultrasound: The Official Journal of the American College of Veterinary Radiology and the International Veterinary Radiology Association 64, no. 6 (2023): 1126–1127.

